# Blood transcriptomics of captive forest musk deer (*Moschus berezovskii*) and possible associations with the immune response to abscesses

**DOI:** 10.1038/s41598-017-18534-0

**Published:** 2018-01-12

**Authors:** Xiaoning Sun, Ruibo Cai, Xuelin Jin, Aaron B. A. Shafer, Xiaolong Hu, Shuang Yang, Yimeng Li, Lei Qi, Shuqiang Liu, Defu Hu

**Affiliations:** 1 Laboratory of Non-invasive Research Technology for Endangered Species, College of Nature Conservation,Beijing Forestry University, No. 35 Tsinghua East Road, Haidian District, Beijing, 100083 China; 2 Shaanxi Institute of Zoology, No. 88 Xing Qing Ave Xian, Shaanxi, 710032 China; 3 Forensic Science and Environmental & Life Sciences, Trent University,1600 West Bank Drive,Peterborough, Ontario, Canada

## Abstract

Forest musk deer (*Moschus berezovskii*; FMD) are both economically valuable and highly endangered. A problem for FMD captive breeding programs has been the susceptibility of FMD to abscesses. To investigate the mechanisms of abscess development in FMD, the blood transcriptomes of three purulent and three healthy individuals were generated. A total of ~39.68 Gb bases were generated using Illumina HiSeq 4000 sequencing technology and 77,752 unigenes were identified after assembling. All the unigenes were annotated, with 63,531 (81.71%) mapping to at least one database. Based on these functional annotations, 45,798 coding sequences (CDS) were detected, along with 12,697 simple sequence repeats (SSRs) and 65,536 single nucleotide polymorphisms (SNPs). A total of 113 unigenes were found to be differentially expressed between healthy and purulent individuals. Functional annotation indicated that most of these differentially expressed genes were involved in the regulation of immune system processes, particularly those associated with parasitic and bacterial infection pathways.

## Introduction

Forest musk deer (*Moschus berezovskii*; FMD) are primarily found in Southern Asia and are economically valuable due to the musk that is secreted by the musk gland of males^1–3^. China has the largest population of FMD and is the source of over 70% of the global musk supply^4^. Extensive illegal hunting and the anthropogenic disturbance of suitable habitats have decimated wild FMD populations in China^3,5–7^. To help save the FMD from extinction, the Chinese government, since 1958, has encouraged enterprises to participate in captive breeding programs^8,9^. These captive breeding programs face serious problems, particularly that of disease^10,11^ with abscesses accounting for 50% of all diagnoses^12,13^. Infections arise when foreign bacteria enter the blood stream and cause suppurative lesions and abscesses in various organs and tissues of the infected animals^14,15^, which there is likely variation in gene expression among health and infected cells. Abscesses can cause any tissues or organs, from the head to the extremities including the mouth or the digestive tract, to become purulent^16^. It is difficult to cure abscesses^10^ and the frequency with which FMD contract the disease has hampered efforts to increase the captive population numbers^17–19^.

Luo *et al*.^20^ suggested that the FMD purulent diseases were primarily caused by *Escherichia coli*. In contrast, Zhao *et al*.^12^ found that *Arcanobacterium pyogenes* (also known as *Trueperella pyogenes*) was the primary aetiological agent of abscesses in FMD, causing secondary and mixed infections, eventually leading to serious illness and death. Another study of bacterial pathogens in FMD found that suppurative disease was typically caused by *Pasteurella* spp. or *Pseudomonas aeruginosa* or *Yersinia* spp., *Actinomyces pyogenes* and *Staphylococcus* spp.^21^. These apparently conflicting results highlight the complexity of the pathogeny of the suppurative disease in FMD^19,22^. To date, quinolone antibiotics, especially ciprofloxacin, are the most effective drugs for treatment of abscesses in FMD^23^.

Very few studies of endangered ungulates have approached understanding disease threats from the molecular level. Two studies have assessed the genetic diversity of captive populations of FMD using small numbers of molecular markers^9,24^. Due to the key role that the major histocompatibility complex (MHC) plays in immune responses, the genetic diversity of the MHC class II proteins was linked to abscesses in FMD^25–27^ and it has been suggested that the MHC plays a critical role in determining the resistance or susceptibility of an individual FMD to abscesses^26^. However, knowledge of the mechanism of abscess formation in FMD is limited, partly due to the lack of understanding of how gene regulation impacts disease formation and progression. This limitation is not trivial because diseases, as we have noted with purulent disease, are usually complex and involve numerous diverse metabolic pathways.

Transcriptomes are the complete set of the mRNAs of a specific tissue or cell at a particular stage or under a given physiological condition. Transcriptome analyses can be used to identify functional elements of the genome, to reveal the molecular constituents of cells and tissues, and to analyze biological processes at the molecular level^28,29^. The immune system is vital for the maintenance of health and plays an important role in the pathogenesis of many diseases. As blood is a major component of the immune system^30,31^, the profiling of blood gene transcripts is often used to analyze the immune response^31^. Moreover, analysis of the blood transcriptome can contribute to the identification of immune pathways, and more broadly has been profiled in several mammals, including brown bears^32^, polar bears^32^, humans^31,33,34^, swine^35^, cattle^36^ and giant panda^37^. Therefore, transcriptome analysis is a powerful means with which to explore disease pathogenesis, physiological homeostasis and the complexity of systems biology^38^.

In this study, we used high-throughput sequencing (HTS) to generate blood transcriptome profiles of 6 captive FMD: 3 purulent and 3 healthy. Through a comparison of the blood transcriptome responses between the two groups, we aimed first to perform a functional annotation of the blood transcriptome of captive FMD, and secondly to identify the signatures that differed between purulent and healthy FMD. Finally, we discuss the role of the FMD immune system and gene regulation in the process of abscesses formation.

## Results

### Sequencing and assembly

To obtain a global overview of the FMD blood transcriptome, 6 cDNA samples from the two groups (healthy and purulent) of FMD were prepared and sequenced on a HiSeq 4000. All short reads have been deposited in the Sequence Read Archive of the National Center for Biotechnology Information (NCBI SRA; accession numbers: SRA 616228). In total, we generated ~39.68 Gb raw sequence reads from the 6 samples with an average of 68.13 Mb from each sample (Supplementary Table S1). After stringent quality assessment and data filtering, we selected 65.75–66.30 Mb high-quality clean reads from each sample for further analysis. All analyzed reads had more than 97.28% Q20 bases (Q20 indicates the rate of bases where quality is greater than 20; see Supplementary Table S1 for an overview). After assembly, we recovered 77,752 unigenes with a total length of 78,169,844 bp, an average length of 1,005 bp, an N50 of 2,206 bp, and a GC content of 51.91% (Table 1).

**Table 1:** Unigenes of the forest musk deer (*Moschus berezovskii*) transcriptome generated in this study.

Sample	Total unigenes	Total length of unigenes (bp)	Mean length of unigenes (bp)	N50 (bp)	N70 (bp)	N90 (bp)	GC (%)
Purulent 1	44097	36962323	838	1549	792	306	51.45
Purulent 2	46283	42021313	907	1736	906	328	51.37
Purulent 3	53331	40364593	756	1357	676	282	51.93
Healthy 1	43475	33762201	776	1384	698	290	52.37
Healthy 2	50759	37704954	742	1312	652	279	52.59
Healthy 3	64862	62759668	967	2052	1041	327	51.64
All-Unigene	77752	78169844	1005	2206	1173	328	51.91

N50/N70/N90: a weighted median statistic where 50%/70%/90% of the total sequence length is contained in unigenes greater than or equal to this value; GC (%): the percentage of G and C bases in all transcripts.

### Transcriptome annotation and CDS prediction

**Figure 1:**
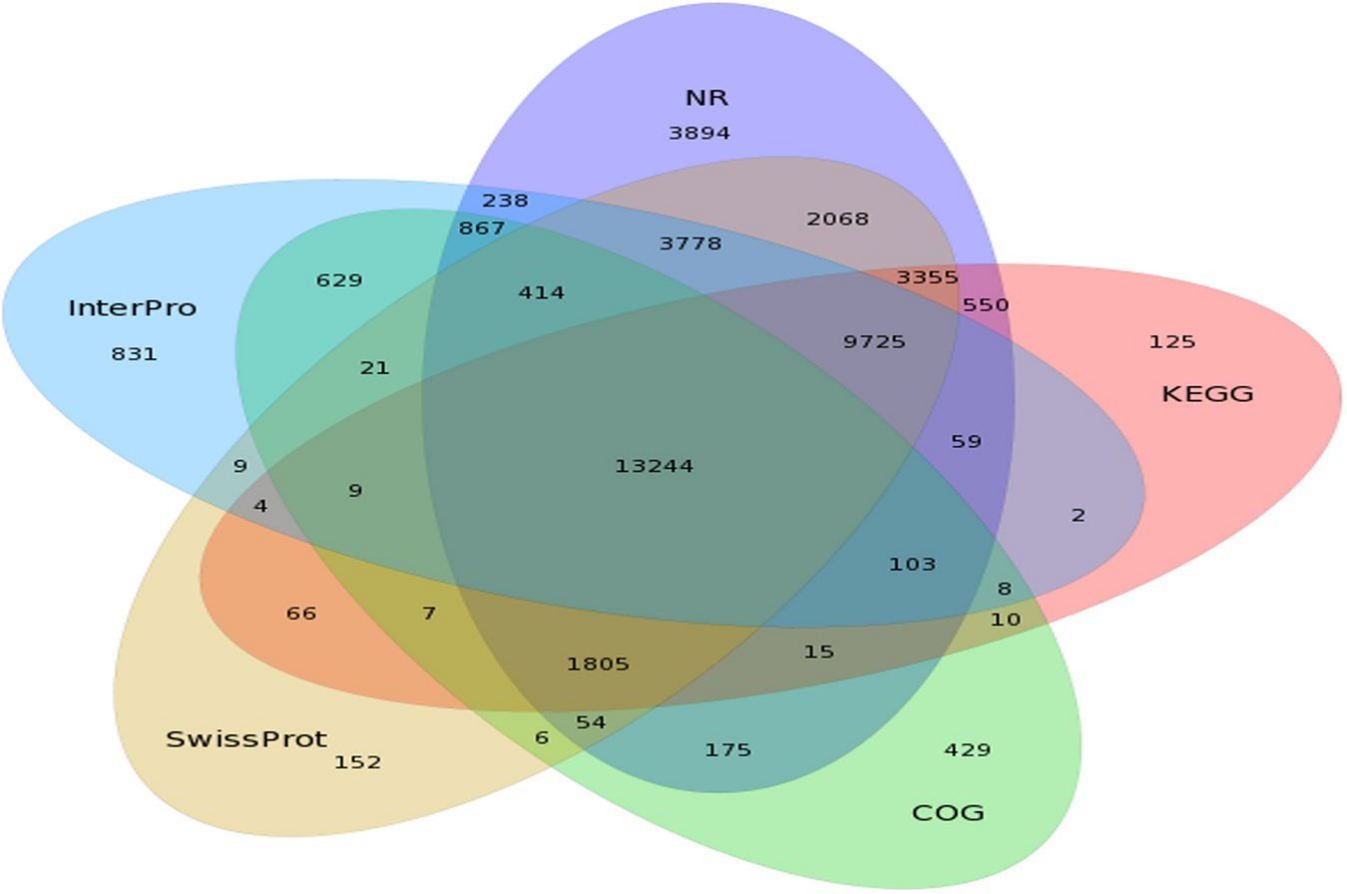
Venn diagram of unigene mapping results. Areas of overlap show the number of unigenes successfully mapped to all overlapping databases. Results are shown only for Non-Redundant protein database (NR), Cluster of Orthologous Groups of proteins(COG), Kyoto encyclopedia of genes and genomes (KEGG), Swiss-prot, and InterPro databases.

We successfully annotated 63,531 (81.71%) of our unigenes with 7 functional databases (Non-Redundant protein database (NR), Nucleotide sequence database (NT), Gene ontology (GO), Cluster of Orthologous Groups of proteins(COG), Kyoto encyclopedia of genes and genomes (KEGG), SwissProt and InterPro; Table 2). Preliminary functional annotations are shown in Supplementary Fig. S2, S3, and S4. Specifically, 58,736 of our unigenes (75.54%) had similar matches in NT, while 17,796 (22.89%) were significantly similar in COG. Concurrently, we analyzed unigenes separately in the NR, COG, KEGG, Swissprot and Interpro databases. We found that 3894 unigenes were matched uniquely in NR, 429 only in COG, 125 only in KEGG, 152 only in SwissProt, and 831 only in InterPro. Only 13,244 of our unigenes (17.03%) had significantly similar matches in all five databases (Fig. 1). Across all unigenes we predicted 45,798 CDS. The predicted CDS had a mean length of 780 bp and an N50 of 1,374 bp. The length of the predicted CDS ranged from 200 bp to more than 3000 bp, up to 36,741 CDS concentrated in 200–1200 bp in length which accounted for 80.24% of unigenes (Supplementary Fig. S5; Supplementary Table S2).

**Table 2:** Percentage of genes that were successfully annotated in each functional database searched.

Item	Number	Percentage
Total unigenes	77,752	100%
NR	40,344	51.89%
NT	58,736	75.54%
SwissProt	34,717	44.65%
KEGG	29,087	37.41%
COG	17,796	22.89%
InterPro	29,941	38.51%
GO	20,510	26.38%
Overall	63,531	81.71%

‘Overall’ indicates the number of unigenes that were annotated by at least one functional database.

### SSR detection and SNP identification

**Figure 2:**
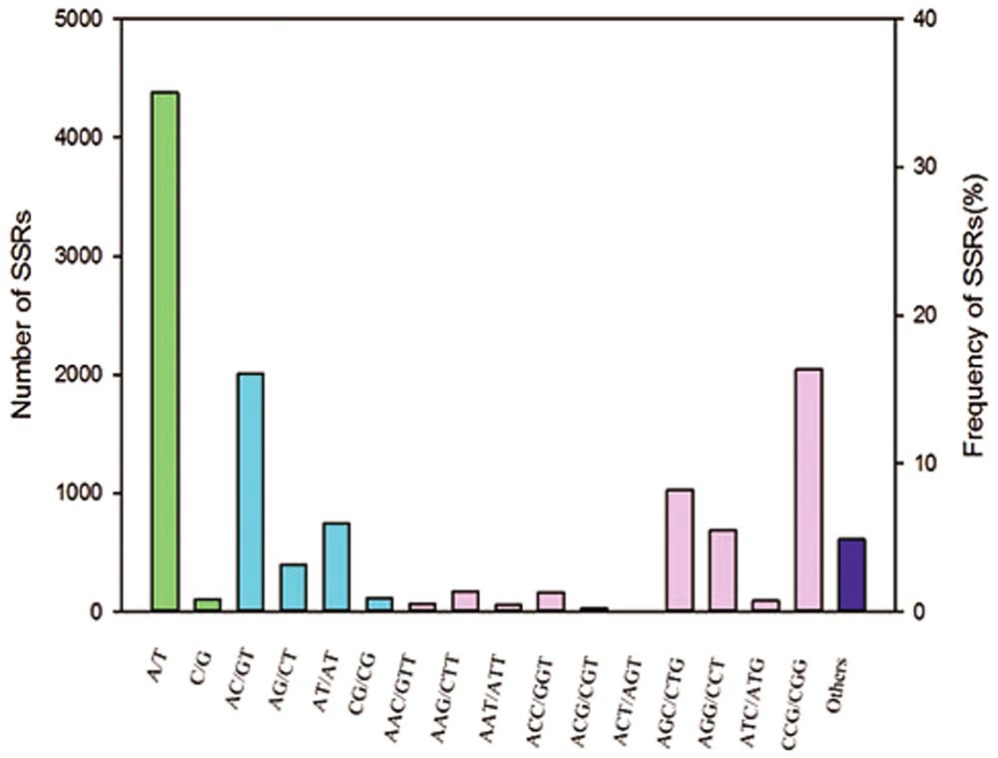
Distribution of potential SSRs by motif type. The 16 primary SSR motifs are shown; the remaining 88 SSR motifs identified are grouped in the “Others” category. Green, light blue, pink and dark blue represent mononucleotide repeats, dinucleotide repeats, trinucleotide repeats and other types of repeats, respectively.

We identified 5,093 potential SSRs in 9,293 unigenes (54.80%); 2,304 of these unigenes contained more than one SSR. Among these SSRs, the most abundant motifs were the mononucleotide repeats (35.26%), the trinucleotide repeats (34.27%), and the dinucleotide repeats (25.68%). Quad-, penta-, and hexanucleotide repeats appeared with lower frequency. The most common motifs were the mononucleotide SSR (A/T)n (42.09 SSR/Mb), dinucleotide SSR (AC/GT)n (19.26 SSR/Mb) and trinucleotide SSR (CCG/CGG)n (19.65 SSR/Mb; Fig. 2; Supplementary Table S3).

We identified 65,526 SNPs across the 6 FMD. For each individual, the ratio of transitions to transversion (Ts/Tv) was between 2.6 and 2.7 (Supplementary Table S4). When comparing SNPs between the 2 groups (healthy and purulent), we found that 770 SNPs were conserved within groups but polymorphic between groups, indicating that variation at these SNPs sites might be closely related to the occurrence of abscesses in FMD (Supplementary Table S6).

### Analysis of DEGs

**Figure 3:**
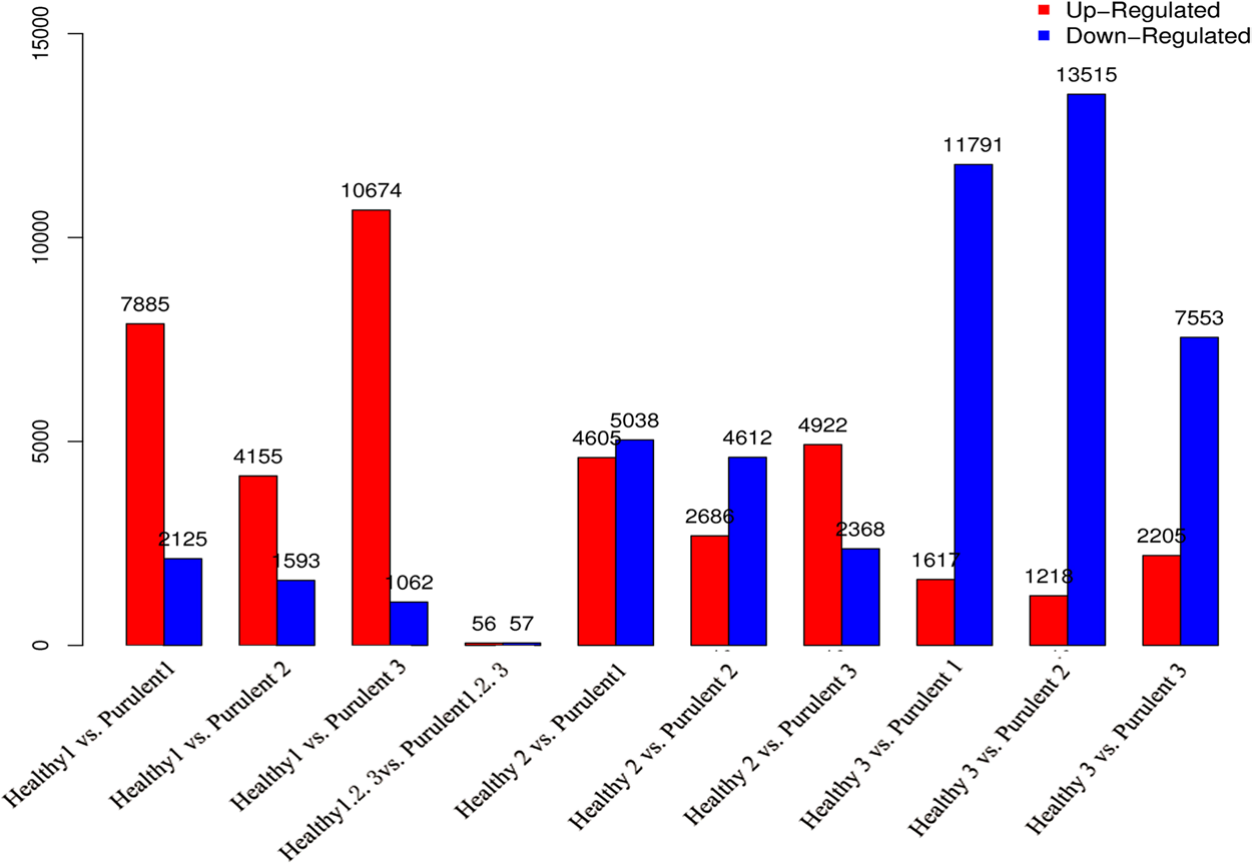
Summary of genes differentially expressed in the healthy and purulent individuals. Each pair of individuals compared is plotted on the x-axis, and the number of differently expressed genes (DEGs) is plotted on the y-axis. The red bars represent the upregulated genes, while the blue bars represent the downregulated DEGs.

We found that 113 of the 77,752 total unigenes (0.15%) were differentially expressed between the two FMD groups: 56 were upregulated in the purulent individuals as compared to the healthy individuals, and 57 were downregulated. PCA analysis of these gene expression patterns showed that DEGs from the healthy FMD clustered separately from the DEGs from the purulent FMD (Fig. 3; Supplementary Table S5; Figure S6).

**Figure 4:**
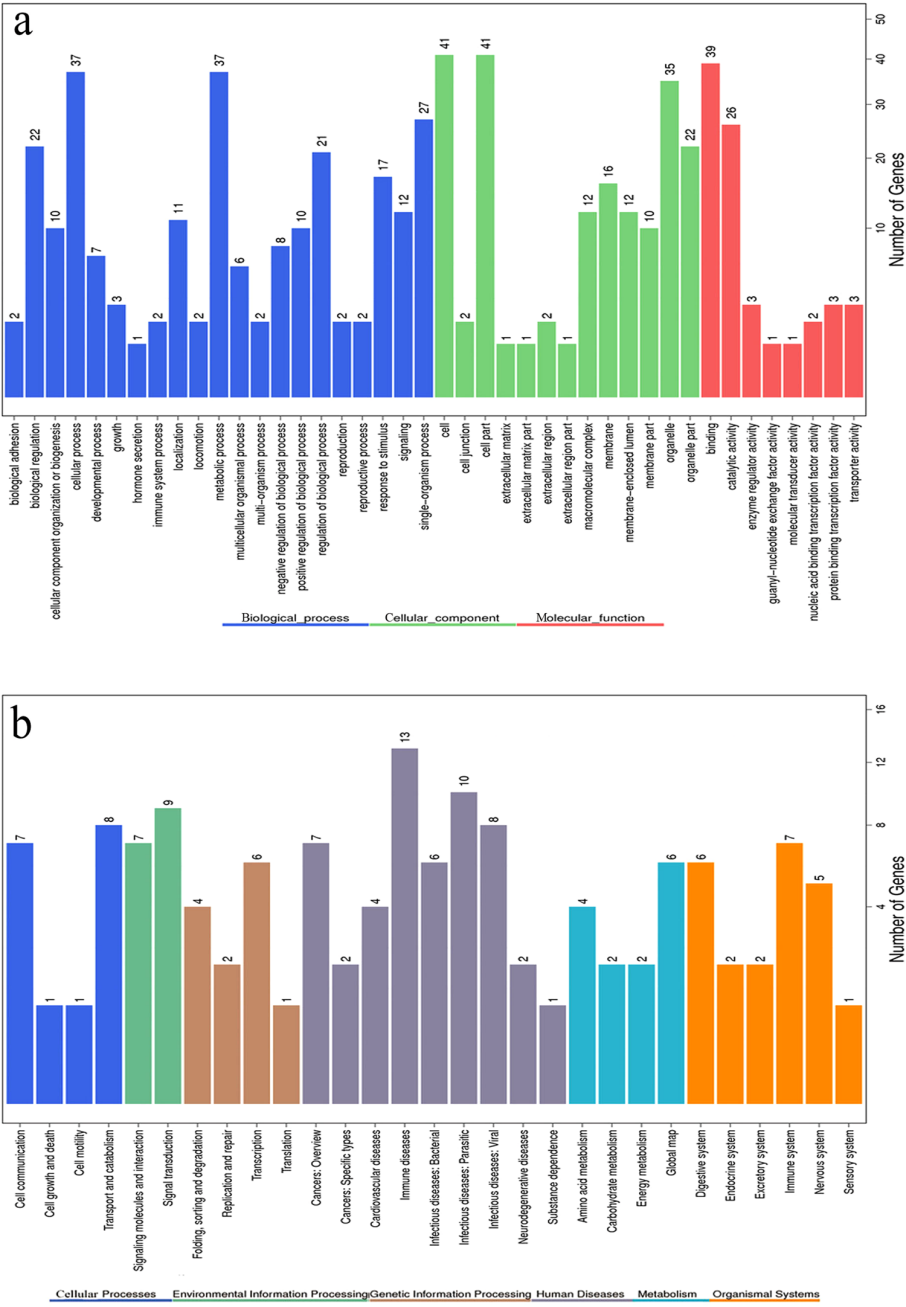
Functional distribution of differently expressed genes (DEGs). (**a**) Functional distribution of DEGs according to the gene ontology (GO) database. The y-axis shows the GO functional categories, while the number of genes in each category is plotted on the x-axis. (**b**) Functional distribution of DEGs according to the KEGG pathway database. The y-axis shows the KEGG functional categories, while the number of genes in each category is plotted on the x-axis.

The GO annotation classified the differentially expressed genes into 3 categories: molecular functions, cellular components and biological processes (Fig. 4a). Molecular functions included genes involved in binding (39 genes; GO:0005488) and catalytic activity (26 genes; GO:0050790). Genes related to cellular components were primarily cell (41 genes; GO:0005623) and cell part (41 genes; GO:0044464) related. Biological process genes were mainly involved in metabolism (37 genes; GO:0008152) and cellular processes (37 genes; GO:0009987). The distribution of GO annotations in different functional categories indicated a substantial diversity of DEGs (Fig. 4a).

We identified biochemical pathways represented by the unigene category. The KEGG annotation of the DEGs suggested that they were distributed in 29 pathways related to cellular processing (17 genes), environmental information processing (16 genes), genetic information processing (13 genes), disease (53 genes), metabolism (14 genes), and organismal systems (23 genes; Fig. 4b). Among the identified functional categories, infectious diseases (24) and immune diseases (13) were most highly represented, followed by immune system (7), signaling molecules and interaction (7), and cancers (9).

### Genes related to the FMD immune system response to abscesses

We found that 16 of the 113 DEGs were involved in the immune system of the FMD (Table 3). Among these DEGs, 11 genes encoding immunoglobulin-like proteins and the interleukin-2 receptor gene (IL2RB; Table 3) were upregulated. In contrast, the B-cell translocation gene 1 (BTG1) and genes encoding the T-cell differentiation antigen CD6, the COMM domain-containing protein gene, and the interferon regulatory factor 2 (IRF2) were downregulated (Table 3). We observed several upregulated FMD immunoglobulin genes in primary immunodeficiency and amoebiasis pathways (Supplementary Fig. S7). Of the 770 SNPs that were polymorphic between groups, 27 were found in genes responsible for immune response (Supplementary Table S7).

**Table 3:** Genes related to immune system function that were differentially expressed in purulent individuals, as compared to healthy individuals.

Gene	Functional annotation	Regulation in purulent samples
Unigene53584_All	immunoglobulin heavy chain variable region precursor, mRNA	Up
CL119.Contig12_All	immunoglobulin light chain variable region (IGLV) mRNA	Up
CL984.Contig6_All	Ig kappa chain, mRNA	Up
Unigene53558_All	immunoglobulin mu heavy chain variable region mRNA	Up
CL465.Contig8_All	mRNA for immunoglobulin heavy chain variable region (ighv gene)	Up
CL6305.Contig5_All	immunoglobulin lambda-like polypeptide 5-like (LOC102250240), mRNA	Up
Unigene4040_All	interleukin 2 receptor, beta (IL2RB), mRNA	Up
Unigene26557_All	immunoglobulin mu heavy chain variable region mRNA	Up
Unigene25688_All	immunoglobulin V lambda chain (V lambda 12.2) gene	Up
Unigene25744_All	immunoglobulin V lambda chain 5.3.6 gene	Up
CL6457.Contig5_All	immunoglobulin lambda-like polypeptide 1-like	Up
CL119.Contig13_All	immunoglobulin V lambda chain (V lambda 12.2) gene	Up
Unigene55659_All	immunoglobulin lambda-like polypeptide 1-like	Up
CL1983.Contig6_All	CD6 molecule (CD6), transcript variant X 4, mRNA	Down
CL4014.Contig3_All	interferon regulatory factor 2 (IRF2), mRNA	Down
CL4718.Contig2_All	B-cell translocation gene 1, anti-proliferative, mRNA	Down

## Discussion

When bacteria or viruses infect organisms patterns of gene expression within cells change dramatically^36,37^. Many of these changes are crucial to the immune response and characterizing patterns of gene expression patterns in a viral or bacterial infection can provide important clues to understanding the cells resistance to pathogen infection. Using blood transcriptomes to study disease has several potential benefits^39^. Firstly, peripheral blood is relatively easier to acquire than the tissue of organism and often causes less damage to the sampled animal, which is particularly relevant for the study of wild animals^37^. Secondly, blood is an important component of the immune system and it is useful to study it directly^31,37^. Thirdly, blood-based profiles have the potential to advance our understanding of disease pathogenesis^30^. Therefore, a comprehensive understanding of the differences between healthy and purulent FMDs in transcriptome levels is indispensable for understanding the pathogenesis of abscesses.

Our study is the first example of the use of Illumina paired-end sequencing technology to investigate the whole blood transcriptome of FMD. To our knowledge, no transcriptome resources for FMD are previously available. Here, we analyzed the de novo transcriptomes of two groups of FMD (healthy and purulent) Our de novo analysis identified 113 unigenes that were differentially expressed between the healthy and purulent groups. Of these DEGs, 16 genes were found related to immune system function, which indicates the diversity of the immune response in FMD to the abscess formation disease.

In addition, HTS has recently been used to identify SSRs and SNPs, and it is considered as a time-saving, highly efficient approach^40–42^, particularly in endangered species^37^, where the number of SNPs and SSRs may be used to estimate the overall genetic variation and thus, the ongoing viability of the species. In the FMD blood transcriptome we identified 65,536 SNPs and 12,697 SSRs, which is comparable to Du *et al*.^37^ that detected 28,925 SNPs and 23,460 potential SSRs in the blood transcriptomes of the endangered giant pandas. Our results not only indicate that this endangered species maintains a considerable genetic variation despite the severe reduction in population size, but also serves as a genetic resource for future research into the population genetics of FMD.

Our analysis of the blood transcriptome of the FMD showed that genes were expressed differently in the healthy and purulent groups. In purulent individuals, 13 genes involved in immune response were upregulated. Interestingly, some of these upregulated genes were involved in primary immunodeficiency pathways as well as in the immune response to viral, bacterial and parasitic diseases (Fig. 4b). These results suggest that abscesses in FMD are likely caused by a multitude of intrinsic and extrinsic factors. Indeed, strict screening of SNPs identified 27 directional mutations that occurred in immune-related genes, suggesting that abscesses in FMD are likely due to genetic deficiency or pathogenic invasion (Table S7). Further study should focus on these immune-related genes, as these may provide useful clues to the mechanisms of abscess development in FMD.

In conclusion, our identification of the DEGs between healthy and purulent FMD provide a framework for future studies of abscess disease in this species. More generally, our work increases the understanding of abscess pathogenesis and immune system function. Further, identification of 27 SNPs support the role of both intrinsic (heritable—SNPs) and extrinsic factors (environmental—DGEs) shaping susceptibility. The data set of assembled FMD unigenes presented here will provide the foundation for other functional and comparative genomic studies and immuno-assay development.

## Methods

### Sample collection and RNA extraction

This study was approved by the Ethics Committee of Beijing Forestry University, Beijing, China; Pien Tze Huang Pharmaceutical Corporation, Zhangzhou, China; and Pien Tze Huang Forest Musk Deer Breeding Center, Shaanxi, China (which managed the FMD we sampled). This study was carried out in accordance with the recommendations of the Institution of Animal Care and the Ethics Committee of Beijing Forestry University. All experimental procedures were performed with the help of a local veterinarian.

Blood samples were collected from 6 different FMDs at the Pien Tze Huang Forest Musk Deer Breeding Center in August 2015. The FMD were categorized as healthy (n = 3) or purulent (with symptoms of abscess; n = 3) according to the medical records provided by the Breeding Center. All FMD sampled were both males with similar age between 3 to 3.5 years old except one purulent male of 7 years old. Individuals in the purulent group contained superficial abscesses. All 6 FMDs were kept in the same captive environment, with the same diet and the same standards of sanitation.

Using disposable sterile syringes we extracted 1–2 mL venous blood from the elbows of each FMD. Fresh blood was collected in tubes containing EDTA and stored in liquid nitrogen. The total RNA of each sample was extracted using TRIzol reagent (Invitrogen, USA) following the manufacturer’s instructions and treated with DNase I (Qiagen, Mississauga, Ontario, Canada). The quality and quantity of total RNA was analyzed using an Agilent 2100 Bioanalyzer, and RNA integrity was checked with agarose gel electrophoresis (1% agarose gel). Suitable RNA samples were selected for cDNA synthesis.

### cDNA library preparation and sequencing

Poly (A) mRNA was isolated using oligo-dT beads. All mRNA was broken into shorter fragments (ca. 200 nt) by adding a fragmentation buffer, and cDNA was synthesized using the mRNA fragments as templates. Short fragments were purified using a QIAquick PCR extraction kit (Qiagen, Germany) and resolved with EB buffer (Qiagen, Germany) for end repair and single nucleotide A (adenine) addition. The short fragments were then ligated with adapters. Suitable fragments were selected for PCR amplification to construct the final cDNA library. The cDNA library was sequenced with RNA sequencing (RNA-Seq) on an Illumina HiSeq 4000 sequencing platform (Illumina HS4000 PE101) using paired-end technology (Fig. S1) at BGI (Shenzhen, Guangdong, China).

### Transcriptomic data analysis

Raw reads were cleaned first by removing all adaptor sequences, followed by removing reads in which unknown bases (N) made up more than 5% of the total sequence, and finally by removing reads where the quality of more than 20% of all bases was less than or equal to 10. After this data filtering, the remaining “clean reads” were assembled *de novo* with Trinity v2.0.6^43^ to produce transcripts. We then clustered these transcript sequences with Tgicl v2.0.6^44^ to generate unigenes. We calculated the length of all unigenes combined as well as average unigene length, N50 (a weighted median statistic where 50% of the total gene length is contained in Unigenes greater than or equal to this value), and GC-content of each unigene.

We used BLAST v2.2.23^45^ with a threshold E-value of 10^−5^ to search for our unigenes in the databases NT (ftp://ftp.ncbi.nlm.nih.gov/blast/db), NR (ftp://ftp.ncbi.nlm.nih.gov/blast/db), COG (http://www.ncbi.nlm.nih.gov/COG), KEGG (http://www.genome.jp/kegg), and SwissProt (http://ftp.ebi.ac.uk/pub/databases/swissprot). We generated gene ontology (GO) annotations for our unigenes with Blast2GO v2.5.0^46^, and used InterProScan5 v5.11–51.0^47^ to generate InterPro (http://www.ebi.ac.uk/interpro) annotations.

We selected the segment of each unigene that best mapped to one or more functional databases as its coding sequence (CDS). Coding regions of unigenes that could not be aligned with any of the functional databases were predicted by ESTScan v3.0.2^48^. We used MISA v1.0^49^ to detect simple sequence repeats (SSRs; also known as microsatellites sequences) in our unigenes, and used GATK v3.4–0^50^ to detect single nucleotide polymorphism (SNP) variants among the individual FMD.

### Analysis of differentially expressed genes (DEGs)

We mapped clean reads to unigenes with Bowtie2 v2.1.0^51^. We calculated the gene expression level of reads that could be uniquely mapped to a gene with RSEM v1.2.12^52^. Gene expression levels were calculated as the number of uniquely mapped fragments per kilobase of exon region per million mappable reads (FPKM). This calculation method eliminates the influence of different gene lengths and varying gene lengths on the measurement of gene expression^53^. The FPKM value, therefore, can be used to compare differences in gene expression among samples.

Using FPKM values, we calculated the differential expression of unigenes among samples with NOIseq and PossionDis^54^. Differences in unigene abundance among samples were calculated based on the ratio of FPKM values. Principal component analysis (PCA) was applied to visualize the differences between the arrays. We identified DEGs between the healthy and purulent groups as those where the absolute value of log2Foldchange was >2.0 and probability ≥0.8 (as calculated by NOIseq) while fold change was ≥2.00 and the FDR (false discovery rate) was ≤0.001 (as calculated by PossionDis). To reduce the risk of overfitting the data, we retained only genes where the expression level of genes from different individuals within the same group was relatively consistent. We classified DEGs according to the GO annotation, and then performed GO functional enrichment using phyper in R^55^. Next, we classified DEGs according to the KEGG annotation result and performed pathway functional enrichment using phyper in R.

### Data availability

All data generated or analyzed during this study are included in this published article (and its Supplementary Information files)^56,57^.

## Electronic supplementary material

supplementary information
