# Supplementary material for: Blood transcriptomics of captive forest musk deer (Moschus berezovskii) and possible associations with the immune response to abscesses
Source: Sci Rep. 2018 Jan 12;8:599. doi: 10.1038/s41598-017-18534-0 (PMC5766596; doi:10.1038/s41598-017-18534-0)

**Blood transcriptomics analysis of captive forest musk deer (*Moschus berezovskii*) and** **possible associations with the immune response to abscesses**

Xiaoning Sun^1,＊^,Ruibo Cai^1,＊^,Xuelin Jin^1^,Aaron B. A. Shafer^2^,Xiaolong Hu^1^,Shuang Yang^1^,Yimeng Li^1^,Lei Qi^1^,Shuqiang Liu^1,^*,Defu Hu^1,^*

^1^ *Laboratory of Non-invasive Research Technology for Endangered Species,College of Nature Conservation,Beijing Forestry University,No. 35 Tsinghua East Road, Haidian District, Beijing 100083, China.*

^2^*Forensic Science and Environmental & Life Sciences, Trent University,Peterborough, ON, Canada.*

∗Correspondence authors.Tel.:+86 10 62338130；+86 10 62338104

E-mail addresses: [hudf@bjfu.edu.cn](mailto:hudf@bjfu.edu.cn) (Defu Hu); [liushuqiang@bjfu.edu.cn](mailto:liushuqiang@bjfu.edu.cn) （Shuqiang Liu）

*＊Co- first author*

**Figures**

**
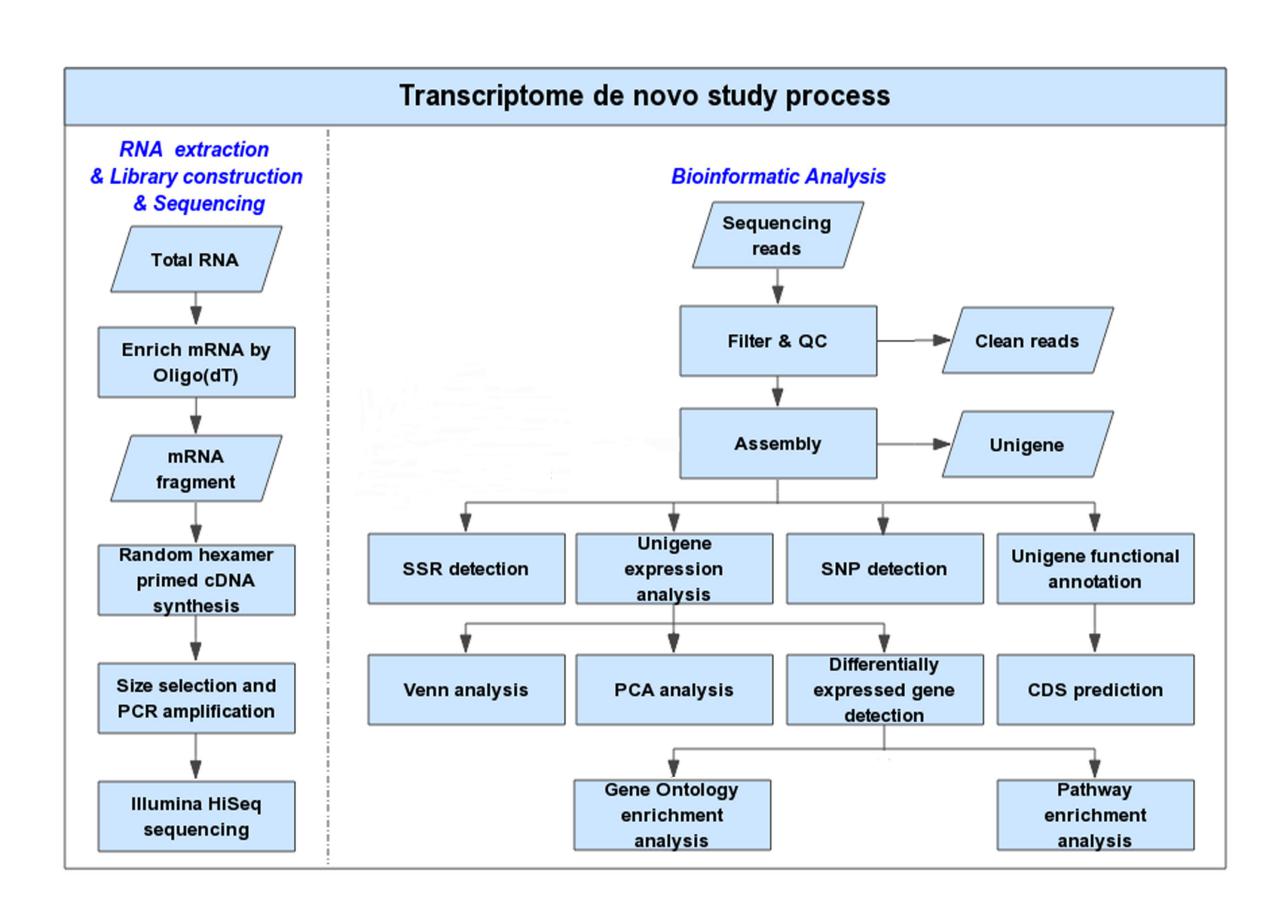
**

**Fig.S1.** **The main steps of the novel transcriptome sequencing process.**

**
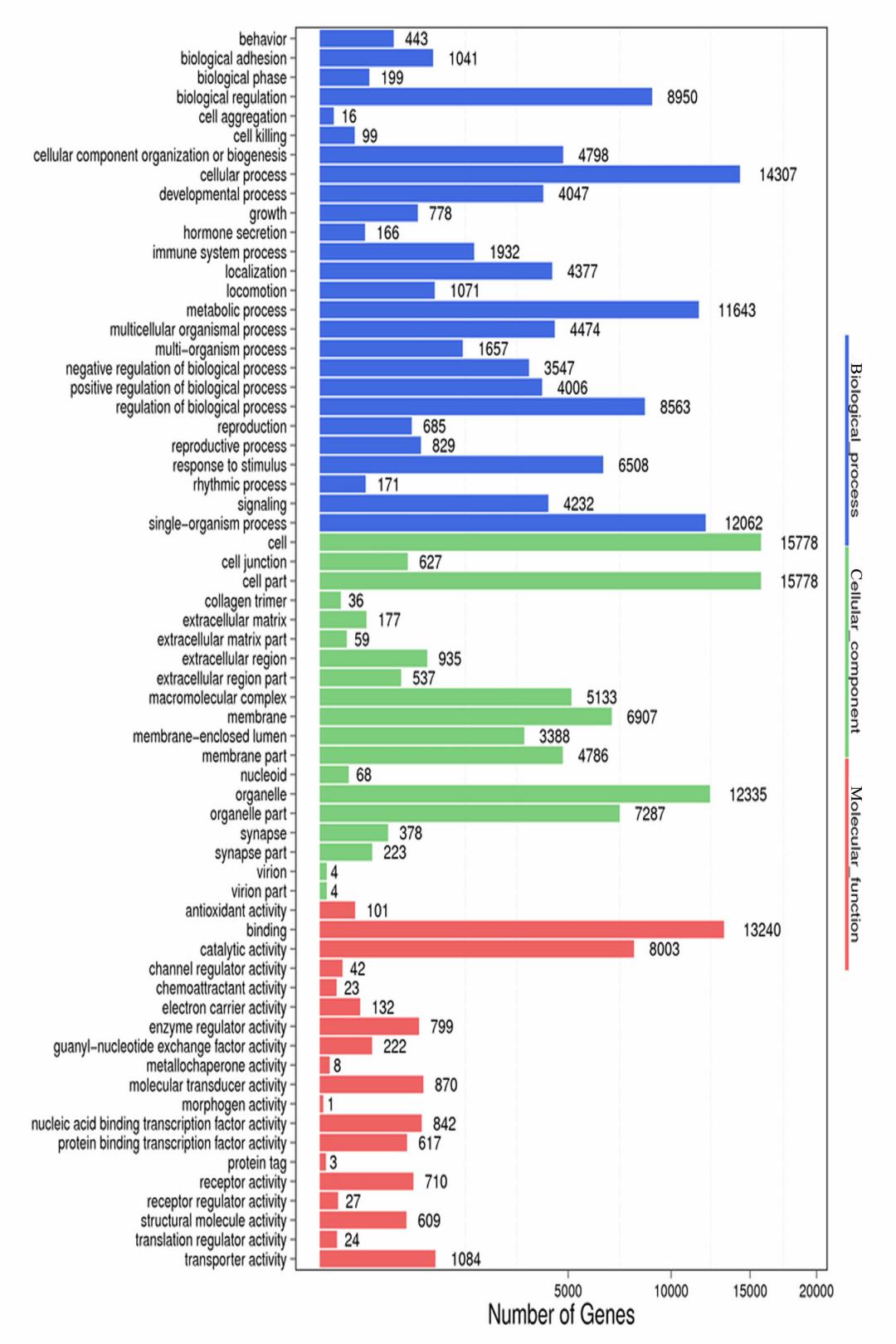
**

**Fig. S2. Functional distribution of GO annotation.** X axis represents the number of Unigenes. Y axis represents the Gene Ontology functional category.


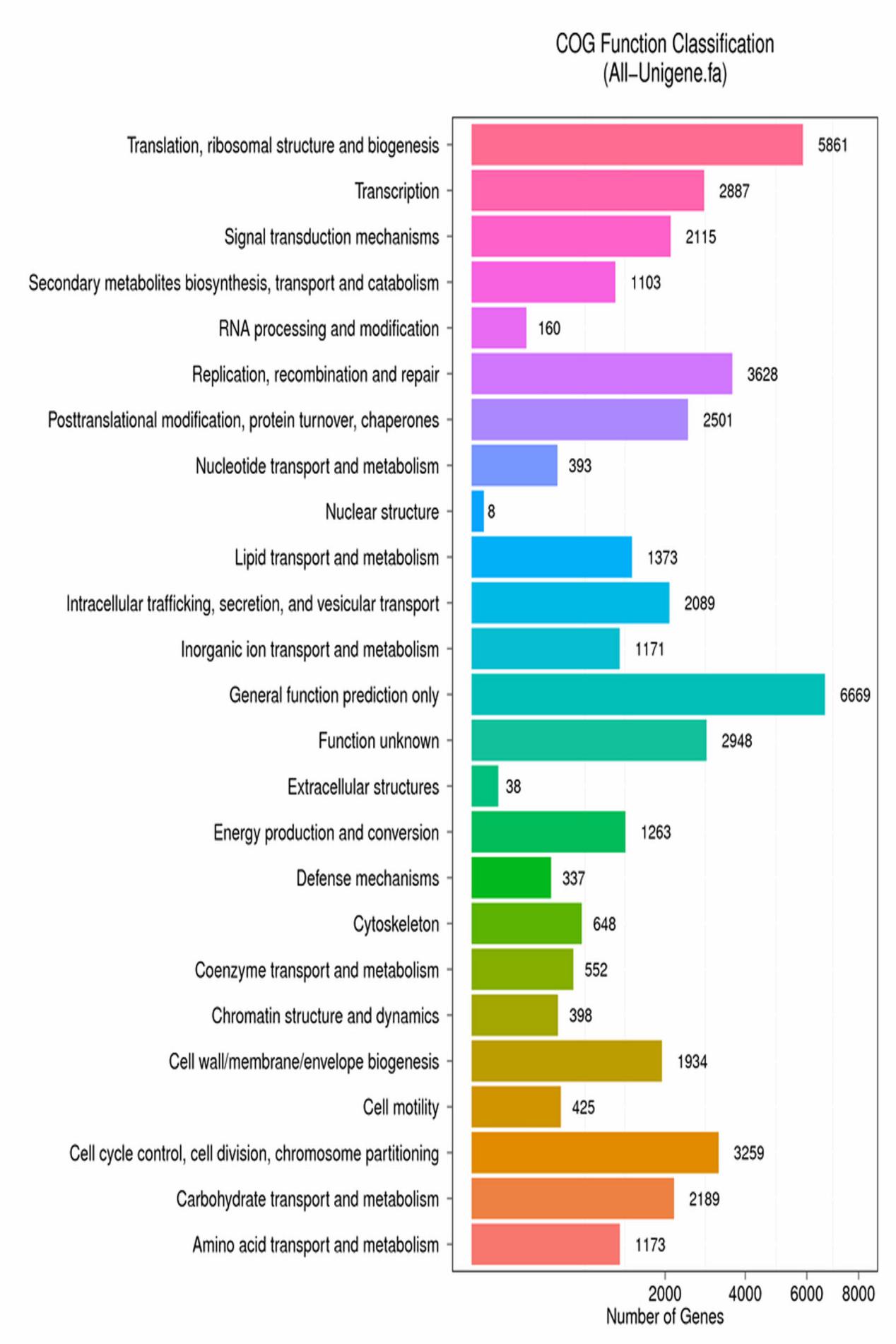


**Fig. S3. Functional distribution of COG annotation.** X axis represents the number of Unigenes. Y axis represents the COG functional category.

**
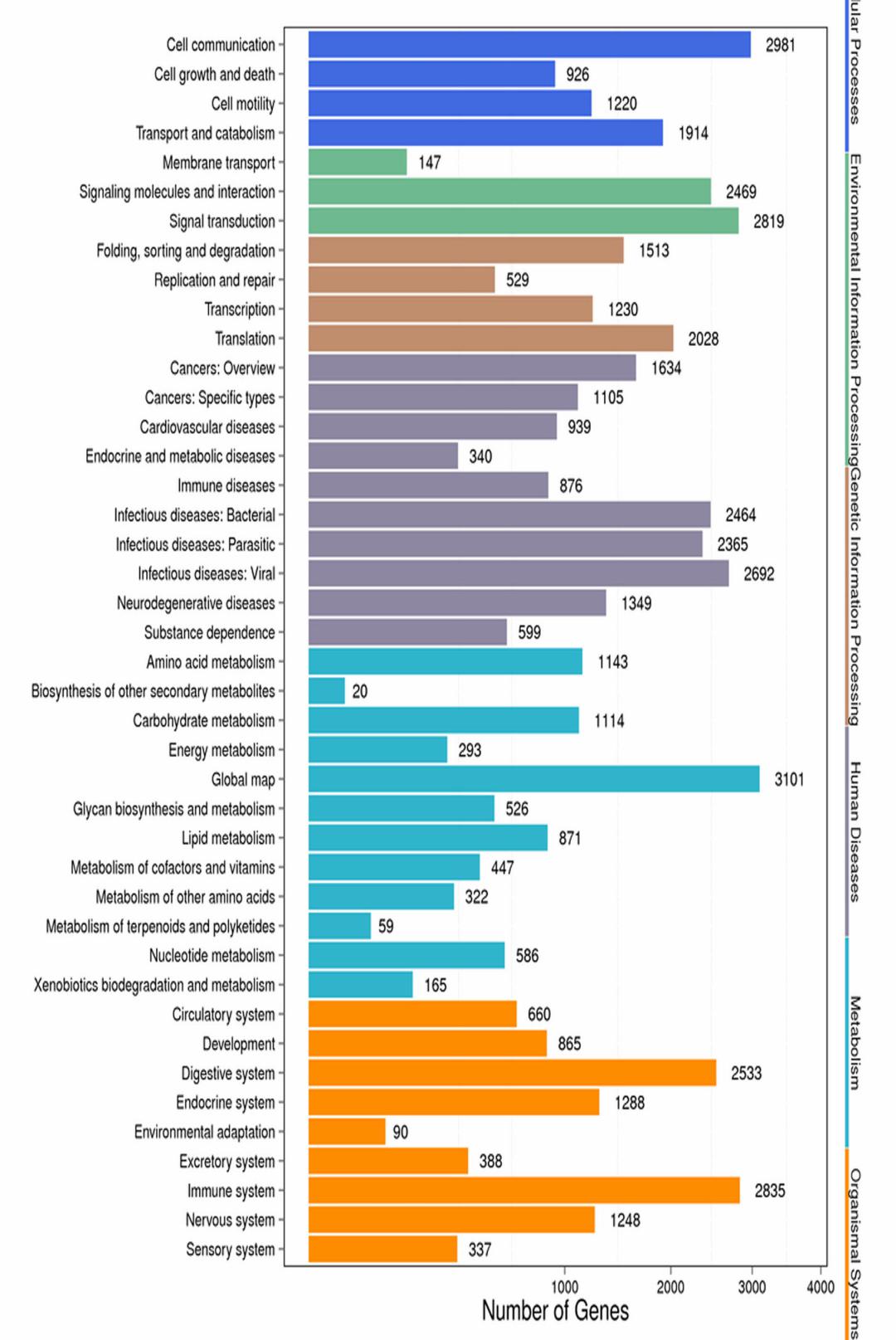
**

**Fig. S4. Functional distribution of KEGG annotation.** X axis represents the number of Unigenes. Y axis represents the KEGG functional category.

**
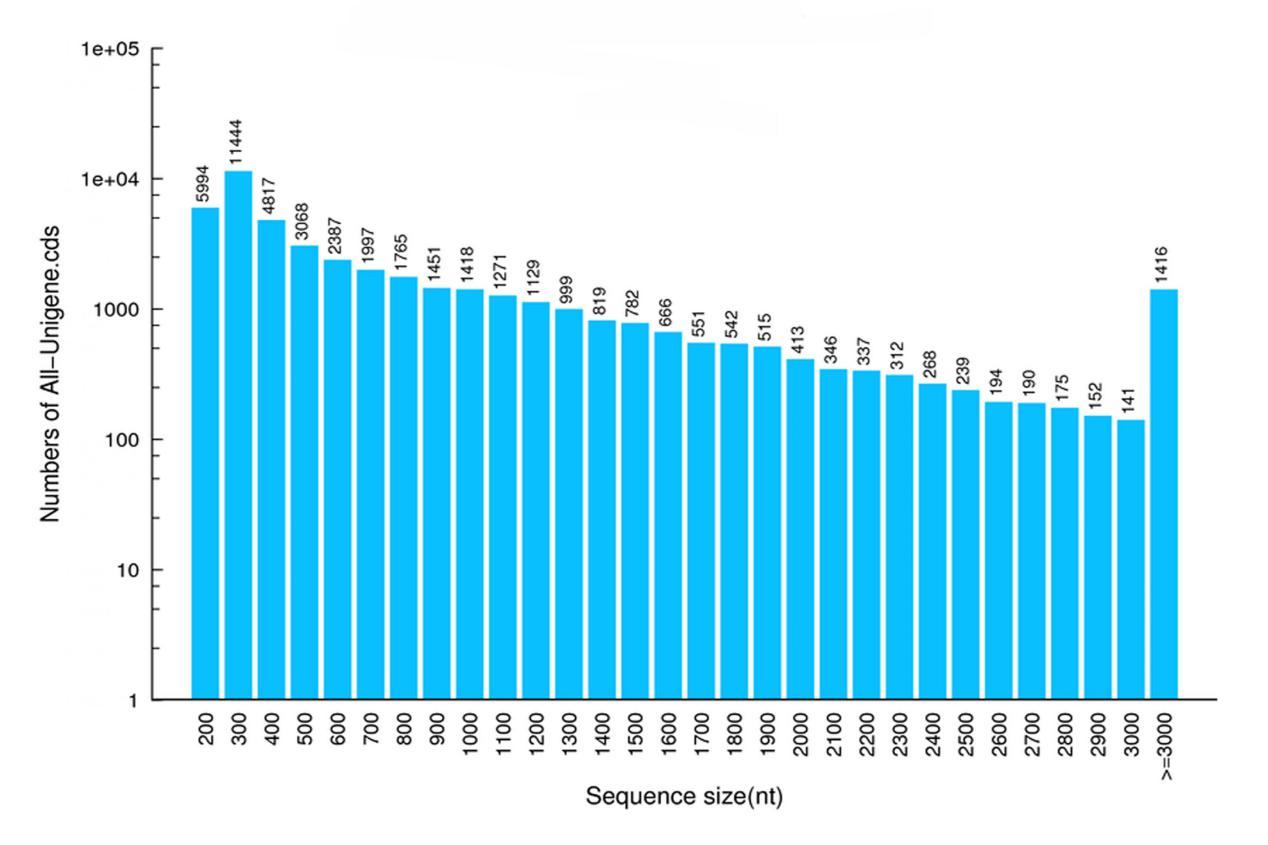
**

**Fig. S5. CDS length distribution**. X axis represents the length of CDS. Y axis represents the number of CDS.


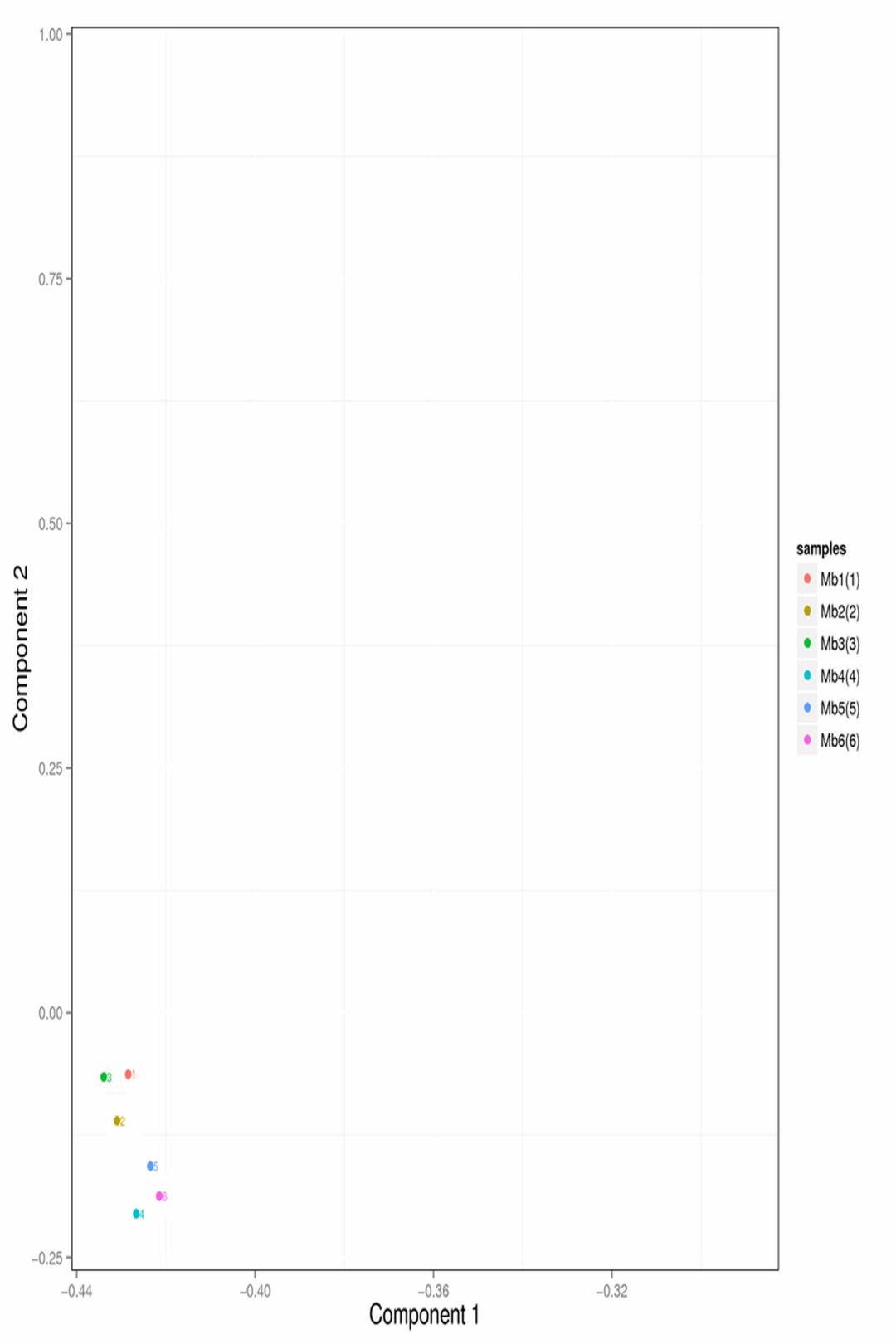


**Fig.S6.** **PCA analysis of six samples**. X axis represents the contributor rate of first component. Y axis represents the contributor rate of second component. Points represent each sample. Mb1, Mb2, Mb3 represents purulent 1,purulent 2,purulent 3,respectively. Mb4, Mb5, Mb6 represents healthy 1,healthy 2 and healthy 3.


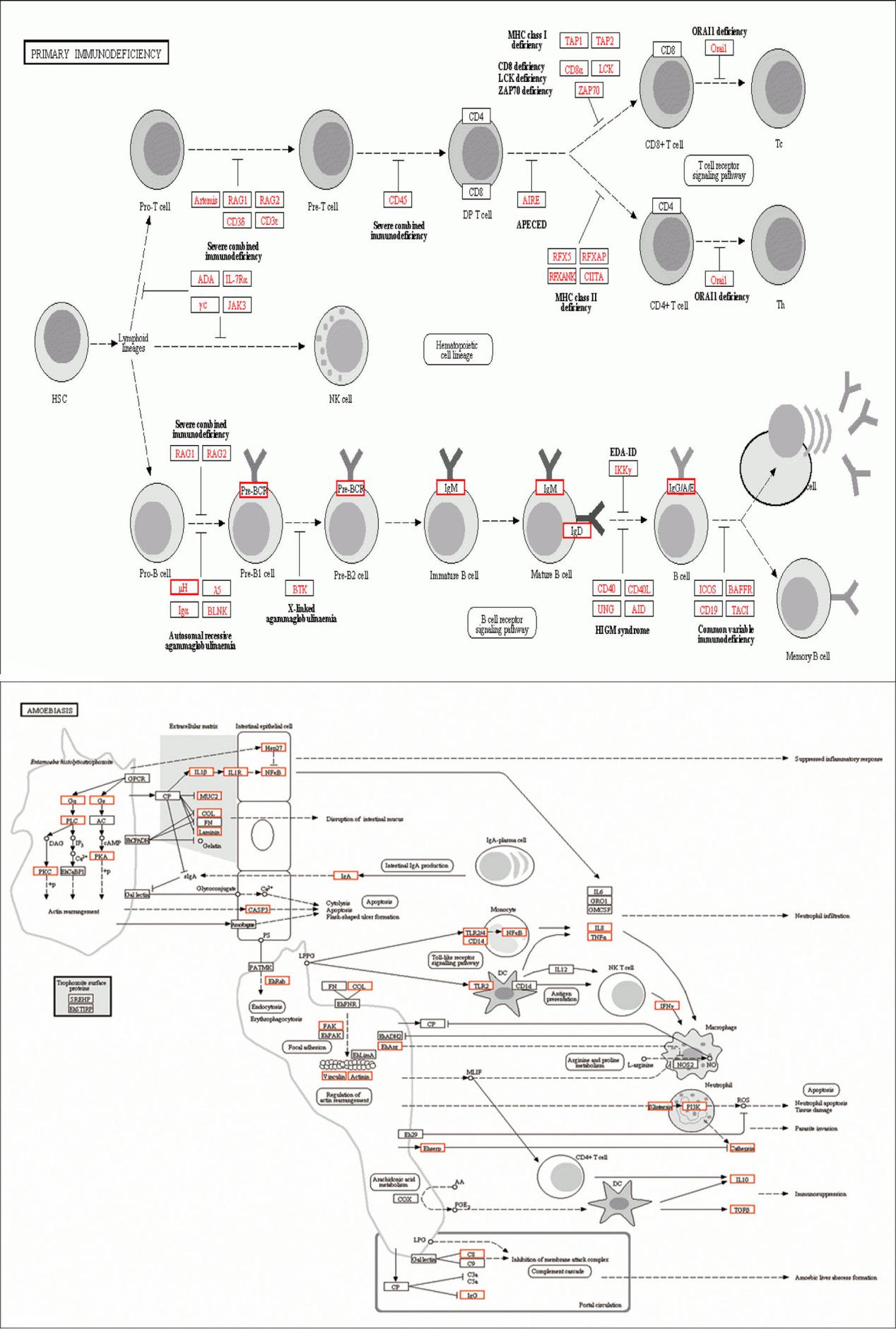


**Fig. S7. Genes in the primary immunodeficiency****^55^ and Amoebiasis pathway significantly expressed ^56^in the FMD transcriptome.** Red boxes indicate the genes that were expressed in the transcriptome.

**Table S1.** Summary of sequencing reads after filtering

| Sample | Total raw reads  (Mb) | Total clean reads  (Mb) | Clean reads Q20  (%) | Clean reads ratio  (%) |
| --- | --- | --- | --- | --- |
| Purulent 1 | 68.13 | 66.06 | 97.43 | 96.96 |
| Purulent 2 | 68.13 | 66.25 | 97.40 | 97.24 |
| Purulent 3 | 68.13 | 65.75 | 97.47 | 96.51 |
| Healthy 1 | 68.13 | 66.20 | 97.56 | 97.16 |
| Healthy 2 | 68.13 | 66.24 | 97.28 | 97.22 |
| Healthy 3 | 68.13 | 66.30 | 97.38 | 97.31 |

**Table S2**. Quality metrics of predicted CDS.N50/N70/N90: a weighted median statistic that 50% / 70% / 90% of the TotalLength is contained in CDS great than or equal to this value;GC (%): the percentage of G and C bases in all CDS.

| Software | Total number | Total length  (bp) | Mean length  (bp) | N50  (bp) | N70  (bp) | N90  (bp) | GC(%) |
| --- | --- | --- | --- | --- | --- | --- | --- |
| Blast | 40953 | 34311180 | 837 | 1434 | 885 | 342 | 55.22 |
| ESTScan | 4845 | 1433592 | 295 | 279 | 237 | 210 | 55.58 |
| Overall | 45798 | 35744772 | 780 | 1374 | 810 | 294 | 55.23 |

**Table S3**. Statistics of SSR detection results for Unigenes in FMD blood transcriptome.

| Searching item | Numbers |
| --- | --- |
| Total number of sequences examined: | 77752 |
| Total size of examined sequences (bp): | 78169844 |
| Total number of identified SSRs: | 12697 |
| Number of SSR containing sequences: | 9293 |
| Number of sequences containing more than 1 SSR: | 2304 |
| Number of SSRs present in compound formation: | 1066 |
| Mono-nucleotide | 4477(35.26%) |
| Di-nucleotide | 3260(25.68%) |
| Tri-nucleotide | 4351(34.27%) |
| Quad-nucleotide | 106(0.83%) |
| Penta-nucleotide | 380(2.99%) |
| Hexa-nucleotide | 123(0.97%) |

**Table S4**. SNP variant type summary.

| Sample | A-G | C-T | Transition | A-C | A-T | C-G | G-T | Transversion | Total | ts/tv |
| --- | --- | --- | --- | --- | --- | --- | --- | --- | --- | --- |
| Purulent 1 | 21380 | 21494 | 42874 | 4213 | 2890 | 4820 | 4306 | 16229 | 59103 | 2.641814 |
| Purulent 2 | 21061 | 20707 | 41768 | 4116 | 2853 | 4792 | 4182 | 15943 | 57711 | 2.619833 |
| Purulent 3 | 19262 | 18923 | 38185 | 3859 | 2632 | 4249 | 3867 | 14607 | 52792 | 2.614158 |
| Healthy 1 | 17191 | 16824 | 34015 | 3332 | 2328 | 3750 | 3368 | 12778 | 46793 | 2.661997 |
| Healthy 2 | 18532 | 18264 | 36796 | 3532 | 2256 | 4135 | 3695 | 13618 | 50414 | 2.702012 |
| Healthy 3 | 24324 | 23988 | 48312 | 4798 | 3299 | 5427 | 4937 | 18461 | 66773 | 2.616976 |

**Table S5.** The list of DEGs between purulent group and healthy group.

| Unigene | log2 Fold Change  (Purulent1.2.3  / Healthy1.2.3) | Probability | Up/Down-Regulation  (Purulent1.2.3  /Healthy1.2.3) |
| --- | --- | --- | --- |
| Unigene28048_All | -8.81485 | 0.912263 | Down |
| CL1769.Contig4_All | -8.42766 | 0.883407 | Down |
| CL1466.Contig4_All | -7.82231 | 0.812677 | Down |
| CL218.Contig21_All | -8.39946 | 0.881243 | Down |
| Unigene4738_All | -9.09628 | 0.928198 | Down |
| Unigene49009_All | -8.00936 | 0.839021 | Down |
| Unigene36739_All | -7.91687 | 0.826464 | Down |
| Unigene45127_All | -8.26515 | 0.983069 | Down |
| CL2591.Contig2_All | -12.5649 | 0.994424 | Down |
| CL1983.Contig6_All | -9.28 | 0.93696 | Down |
| Unigene45826_All | -12.9781 | 0.99585 | Down |
| Unigene10672_All | -7.88671 | 0.822259 | Down |
| CL2418.Contig5_All | -8.02975 | 0.841196 | Down |
| CL2.Contig3_All | -7.76377 | 0.803725 | Down |
| CL2482.Contig1_All | -9.51964 | 0.94716 | Down |
| CL163.Contig5_All | -7.87242 | 0.819827 | Down |
| CL2833.Contig5_All | -7.92085 | 0.826464 | Down |
| CL2962.Contig2_All | -7.93664 | 0.828911 | Down |
| Unigene48786_All | -8.02422 | 0.840507 | Down |
| CL4014.Contig3_All | -7.78136 | 0.806806 | Down |
| CL183.Contig12_All | -8.40656 | 0.881633 | Down |
| CL4452.Contig2_All | -8.44708 | 0.885185 | Down |
| Unigene28568_All | -8.00375 | 0.838025 | Down |
| Unigene6120_All | -8.11894 | 0.852172 | Down |
| CL2016.Contig1_All | -9.189 | 0.932895 | Down |
| CL4718.Contig2_All | -8.60636 | 0.987562 | Down |
| CL2591.Contig1_All | -12.3369 | 0.993453 | Down |
| Unigene48203_All | -8.37649 | 0.878888 | Down |
| CL849.Contig17_All | -8.2854 | 0.870355 | Down |
| CL2285.Contig1_All | -8.19476 | 0.860819 | Down |
| CL2247.Contig4_All | -8.71195 | 0.905578 | Down |
| CL4452.Contig1_All | -6.03873 | 0.808766 | Down |
| CL3377.Contig3_All | -9.56669 | 0.948951 | Down |
| CL1026.Contig6_All | -7.89482 | 0.823081 | Down |
| CL3688.Contig4_All | -8.55587 | 0.894218 | Down |
| Unigene57022_All | -7.86831 | 0.819827 | Down |
| CL952.Contig6_All | -9.25896 | 0.935975 | Down |
| Unigene56364_All | -7.8095 | 0.810785 | Down |
| CL2071.Contig9_All | -8.99435 | 0.922831 | Down |
| CL2482.Contig2_All | -11.8095 | 0.990482 | Down |
| CL6440.Contig5_All | -7.90489 | 0.824598 | Down |
| Unigene47707_All | -12.3373 | 0.993453 | Down |
| CL1936.Contig2_All | -7.75043 | 0.801609 | Down |
| CL1277.Contig4_All | -6.41785 | 0.805738 | Down |
| CL3057.Contig1_All | -7.94447 | 0.829678 | Down |
| Unigene48617_All | -14.0755 | 0.998011 | Down |
| CL1680.Contig5_All | -6.66157 | 0.826102 | Down |
| CL206.Contig1_All | -8.25267 | 0.867033 | Down |
| CL483.Contig14_All | -7.86625 | 0.818904 | Down |
| CL8.Contig23_All | -7.81164 | 0.811752 | Down |
| CL3297.Contig1_All | -8.42066 | 0.883078 | Down |
| Unigene47810_All | -7.78354 | 0.806806 | Down |
| CL2528.Contig1_All | -8.31741 | 0.873602 | Down |
| Unigene47462_All | -9.13725 | 0.978189 | Down |
| CL681.Contig2_All | -7.53008 | 0.857254 | Down |
| CL1147.Contig3_All | -7.79008 | 0.807784 | Down |
| Unigene3431_All | -7.82655 | 0.813695 | Down |
| Unigene55196_All | 8.564785 | 0.895047 | Up |
| CL984.Contig6_All | 7.860052 | 0.818021 | Up |
| CL5638.Contig1_All | 7.874469 | 0.820373 | Up |
| Unigene55176_All | 7.759333 | 0.803725 | Up |
| CL1161.Contig2_All | 8.377934 | 0.879278 | Up |
| CL513.Contig1_All | 10.66711 | 0.977349 | Up |
| CL236.Contig1_All | 8.001877 | 0.837486 | Up |
| CL3472.Contig3_All | 9.130999 | 0.930062 | Up |
| CL3788.Contig3_All | 8.466246 | 0.886886 | Up |
| CL218.Contig25_All | 7.922832 | 0.827212 | Up |
| CL248.Contig8_All | 8.821243 | 0.912633 | Up |
| CL878.Contig1_All | 8.416446 | 0.882742 | Up |
| CL465.Contig8_All | 7.790077 | 0.807784 | Up |
| Unigene26557_All | 8.214319 | 0.862876 | Up |
| Unigene25688_All | 8.060696 | 0.845311 | Up |
| CL6305.Contig5_All | 8.251088 | 0.867033 | Up |
| CL2168.Contig2_All | 9.286173 | 0.937151 | Up |
| Unigene53466_All | 7.912889 | 0.825668 | Up |
| CL6457.Contig5_All | 6.528462 | 0.880681 | Up |
| CL24.Contig2_All | 7.770389 | 0.804903 | Up |
| Unigene53449_All | 7.884679 | 0.944803 | Up |
| CL162.Contig2_All | 8.144658 | 0.855037 | Up |
| CL1832.Contig2_All | 9.033423 | 0.924986 | Up |
| CL1701.Contig3_All | 7.822305 | 0.812677 | Up |
| CL5885.Contig5_All | 8.54818 | 0.89356 | Up |
| Unigene53558_All | 7.774787 | 0.805902 | Up |
| Unigene4040_All | 8.256445 | 0.913938 | Up |
| CL3536.Contig3_All | 8.17825 | 0.859157 | Up |
| CL2554.Contig1_All | 9.011227 | 0.923798 | Up |
| CL5.Contig2_All | 6.50057 | 0.823596 | Up |
| Unigene11540_All | 8.025693 | 0.895192 | Up |
| CL3949.Contig1_All | 7.750428 | 0.801609 | Up |
| Unigene27675_All | 8.356085 | 0.877219 | Up |
| CL3266.Contig3_All | 7.779172 | 0.806806 | Up |
| CL4145.Contig3_All | 8.127564 | 0.853389 | Up |
| Unigene55659_All | 9.684164 | 0.953242 | Up |
| CL1466.Contig5_All | 8.080373 | 0.848004 | Up |
| CL2234.Contig3_All | 7.990577 | 0.836043 | Up |
| CL5045.Contig2_All | 9.466926 | 0.945063 | Up |
| Unigene12470_All | 8.506095 | 0.948258 | Up |
| Unigene53584_All | 7.878562 | 0.820518 | Up |
| CL3256.Contig1_All | 7.824428 | 0.813353 | Up |
| CL976.Contig3_All | 8.396605 | 0.880826 | Up |
| CL2712.Contig3_All | 8.471675 | 0.887228 | Up |
| CL2031.Contig1_All | 7.787903 | 0.807784 | Up |
| CL6229.Contig2_All | 11.28771 | 0.985786 | Up |
| CL119.Contig13_All | 9.960967 | 0.961861 | Up |
| CL6375.Contig2_All | 7.413628 | 0.841126 | Up |
| CL2866.Contig1_All | 8.003752 | 0.838025 | Up |
| CL1003.Contig8_All | 7.807355 | 0.810785 | Up |
| CL4536.Contig1_All | 9.158189 | 0.93122 | Up |
| CL2001.Contig1_All | 7.927185 | 0.88561 | Up |
| CL1483.Contig4_All | 8.342815 | 0.87604 | Up |
| Unigene25744_All | 8.073249 | 0.846706 | Up |
| CL5625.Contig2_All | 8.533979 | 0.892541 | Up |
| CL119.Contig12_All | 7.864186 | 0.818904 | Up |

**Table S6**. Screened SNPs between healthy and purulent groups (see Supplementary Dataset).

**Table S7**. Screened SNPs sites associated with immune response .Black font color indicate validated SNP;Red font color indicate sample-sepcific validated SNP; Green font color indicate invalidated SNP;Blue font color indicate non-SNP;Asterisk indicate no reads covered.

| Unigene | Mb1 | Mb2 | Mb3 | Mb4 | Mb5 | Mb6 |
| --- | --- | --- | --- | --- | --- | --- |
| CL6457.Contig5_All | A | G | A | A | A | A |
| CL6457.Contig5_All | C | A | A | * | * | * |
| CL6457.Contig5_All | C | T | T | * | * | * |
| CL6457.Contig5_All | C | G | C | G | C | G |
| CL6457.Contig5_All | T | T | T | G | T | T |
| CL6229.Contig2_All | T | C | T | T | * | T |
| CL6229.Contig2_All | A | C | A | A | A | A |
| CL119.Contig13_All | T | * | * | * | * | * |
| CL2168.Contig2_All | A | C | C | C | C | C |
| CL2168.Contig2_All | A | G | G | G | G | G |
| CL2168.Contig2_All | T | * | * | * | * | * |
| CL2482.Contig2_All | * | * | * | * | C | * |
| CL2482.Contig2_All | * | * | * | * | A | * |
| CL2482.Contig2_All | * | * | * | * | A | C |
| CL2591.Contig2_All | * | * | * | * | C | * |
| CL2591.Contig2_All | * | * | * | * | A | * |
| CL2591.Contig1_All | * | * | * | * | G | * |
| CL1277.Contig4_All | T | * | * | C | T | T |
| CL2482.Contig1_All | * | * | * | * | T | * |
| CL2482.Contig1_All | * | * | * | * | A | * |
| CL681.Contig2_All | G | G | G | G | A | G |
| CL2962.Contig2_All | T | T | T | T | T | C |
| CL483.Contig14_All | * | * | G | G | A | G |
| CL4718.Contig2_All | G | T | G | G | G | G |
| CL4718.Contig2_All | G | T | G | G | G | G |
| CL2247.Contig4_All | C | C | C | C | C | T |
| CL2285.Contig1_All | * | A | A | * | * | G |


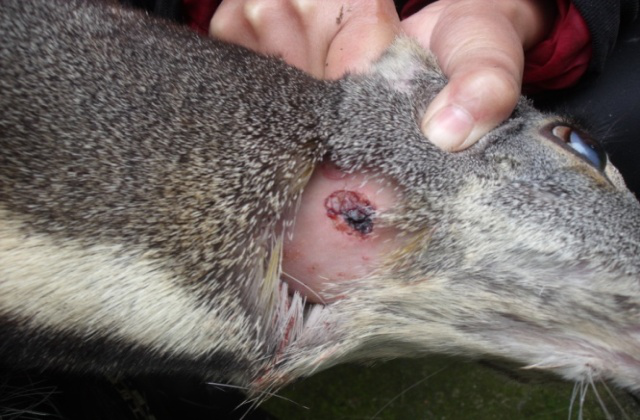


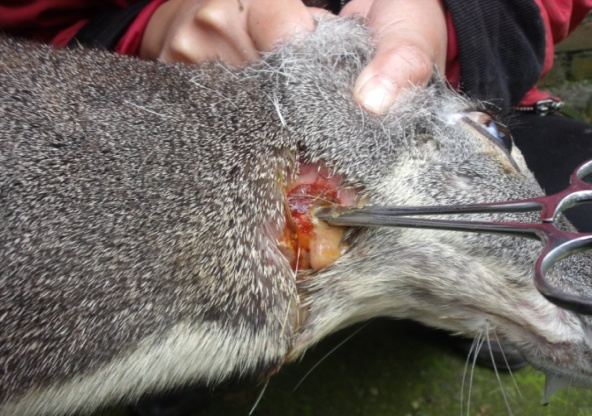

Supplement: Supplementary file 1 — supplementary information [file 41598_2017_18534_MOESM1_ESM.docx]
